# Social Cognition in Rehabilitation Context: Different Evolution of Affective and Cognitive Theory of Mind in Mild Cognitive Impairment

**DOI:** 10.1155/2020/5204927

**Published:** 2020-01-04

**Authors:** Federica Rossetto, Francesca Baglio, Davide Massaro, Margherita Alberoni, Raffaello Nemni, Antonella Marchetti, Ilaria Castelli

**Affiliations:** ^1^IRCCS Fondazione Don Carlo Gnocchi, Milan, Italy; ^2^Research Unit on Theory of Mind, Department of Psychology, Università Cattolica del Sacro Cuore, Milan, Italy; ^3^Department of Pathophysiology and Transplantation, Università degli Studi di Milano, Milan, Italy; ^4^Department of Human and Social Science, Università degli Studi di Bergamo, Bergamo, Italy

## Abstract

Maintaining social skills such as Theory of Mind (ToM) competences is important to counteract the conversion into dementia in Mild Cognitive Impairment (MCI). Multidimensional nonpharmacological interventions demonstrated their potential in improving cognitive and behavioral abilities; however, little is known about the long-term effect of such interventions on social skills in people with MCI. The aim of this longitudinal study was to monitor ToM competences considering both cognitive and affective domains in an amnestic MCI (aMCI) sample involved in a home-based multistimulation treatment (MST@H). 30 aMCI subjects (M : F = 15 : 15; mean age ± SD = 77.00 ± 4.60) were enrolled, and three steps of evaluation with neuropsychological tests and ToM tasks have been implemented. 21 healthy controls (HC) were also included (M : F = 9 : 12; mean age ± SD = 74.95 ± 3.88) to characterize the aMCI sample regarding differences in ToM performance compared to HC at the baseline evaluation. Our results show that the aMCI group statistically significantly underperformed the HC group only in the advanced ToM tasks, confirming an initial decline of high-level ToM competences in this population. The longitudinal evaluation revealed time changes not only in some subcognitive domains of MoCA (memory and executive functions) but also in cognitive and affective ToM dimensions in aMCI subjects. Our findings suggest that cognitive and affective ToM can be considered useful outcome measures to test the long-term effect of treatment over time.

## 1. Introduction

Social cognition refers to the psychological processes that allow individuals to make inference about other people in the context of social interaction [1]. It represents a crucial competence for dealing with our interpersonal relationships in everyday life, by enabling us to anticipate and interpret other's behaviors. In recent years, there has been a substantial increase in the number of studies that investigate social cognitive functions in neurodegenerative diseases, due to a greater awareness concerning the critical role of social cognition in functional and cognitive disability [2]. Recently, the American Psychiatric Association introduced social cognition as one of the six core neurocognitive domains in the latest edition of the Diagnostic and Statistical Manual for Mental Disorders (DSM-5), upholding the importance of the clinical assessment of social cognitive function in several mental disorders, in addition to the conventional neuropsychological assessment [3].

One of the key components of social cognition is Theory of Mind (ToM) [4], which refers to our ability to understand one's own and other's cognitive (thoughts, beliefs, and intentions: “cognitive ToM”) and affective (emotions or feelings: “affective ToM”) states and to predict other's behaviors on the basis of such mental representations. It is now well-documented that failures in such different dimensions of ToM function, such as cognitive and affective components, may represent a core feature of many clinical conditions, leading to impairments in social functioning and poor quality of life [5]. A growing body of research suggests that ToM performance may be used as a screening tool for differentiating between successful and unsuccessful aging (for example, see [6]) and between different forms of neurodegenerative conditions [7–9]. Moreover, the assessment of ToM competences provides the opportunity to monitor the disease progression [5]. This is particularly helpful in Alzheimer's disease (AD), a neurodegenerative condition which represents the most common cause of dementia according to the World Health Organization (WHO) [10]. AD is actually defined as a *continuum* of pathology between elderly individuals with Mild Cognitive Impairment (MCI) and people with frank dementia [11]. With the progressive neurodegeneration, many AD patients show impaired social functioning in addition to cognitive, functional, and behavioral problems. Results indicated that cognitive aspects of ToM are more sensitive to AD progression than affective tasks [12]. Specifically, high levels of cognitive ToM seem to be impaired, especially in the second level of recursive thinking, while the ability to attribute beliefs remains intact at a more basic level (first-order tasks) [13, 14]. The importance of the cognitive decline for ToM abilities is still an open matter of debate, and the association between ToM and cognitive functioning has yet to be deeply explored in AD *continuum.*

There is no medical treatment able to stop or slow down the progression of dementia. Therefore, researchers have recently shifted their attention to nonpharmacological treatment approaches to prevent and treat cognitive deficits [15]. Results of a growing number of studies converge on the effectiveness that these approaches have at the cognitive and behavioral level of people with cognitive impairment (for a review, see [16]). However, these kinds of interventions show two main weaknesses: sustainability and the lack of knowledge concerning the long-term effects. Regarding the first weakness, sustainability in terms of both cost effect and mobility or travel expenses access to care, new ongoing interventions to be delivered at home such as telerehabilitation are now provided. A recent review showed some evidence suggesting that these telerehabilitation programs for people with MCI, Alzheimer's disease, and frontotemporal dementia may have comparable effects as conventional face-to-face cognitive rehabilitation [17]. As concerns the second weakness, the effectiveness of treatment maintenance over time, the available data are only preliminary. Orrell and colleagues have demonstrated cognitive benefits over approximately 8 months [18] with multistimulation treatment, but the long-term effect on social cognitive competences is still unclear and no data are available on telerehabilitation programs.

In this framework, the aim of our study was to investigate, in a longitudinal perspective, potential long-term effects (after 12 months from enrolment) of the home-based multistimulation treatment on social cognitive domain and cognitive functioning in an aMCI sample. The monitoring of the ToM performance, a key component of social cognition, in AD *continuum* in a longitudinal perspective could be a longitudinal outcome of long-term clinical and functional *status* [5], given the intrinsic association between ToM and cognitive functions [19, 20].

## 2. Materials and Methods

### 2.1. Participants

Thirty outpatients diagnosed with aMCI were consecutively recruited from the Memory Clinic of IRCCS Don C. Gnocchi Foundation (see Table 1 for demographic details).

All participants had to meet the following inclusion criteria: (1) diagnosis of MCI due to AD made by the neurologist according to the recommendations of the National Institute on Aging [21] and the DSM-5 diagnostic criteria [22]; (2) age over 65 years and school attendance ≥ 5 years; (3) normal general cognitive function, as determined by the Montreal Cognitive Assessment test [23] (MoCA test score ≥ 15.50), corrected for age and years of education according to Italian normative data [24]; (4) abnormal memory function confirmed by an informant and documented over time by at least three consecutive steps of neuropsychological examination [21, 25]; (5) no impairment in functional activities of daily living as determined by a clinical interview with both the patient and the caregiver; (6) willingness to participate in the MST@H as postdiagnostic care program for aMCI outpatients [26]; (7) absence of psychiatric illnesses, with particular attention to exclusion of participants with a history of depression (Hamilton Depression Rating Scale score ≤ 12) [27] and severe behavioral disturbance; and (8) absence of severe auditory/visual loss.

Twenty-one healthy controls (HC), who were matched for age, education, and gender to the aMCI subjects (see Table 1 for demographic details), were also included in the study for the baseline comparisons, in order to determine the starting level of aMCI subjects with respect to ToM performance. They were screened according to their clinical history in order to exclude major systemic, psychiatric, or neurological illnesses. In particular, the exclusion criteria for HC participants were (1) the presence of visual or auditory deficits; (2) a positive history of psychiatric disorders or behavioral problems; (3) the presence of neurological conditions, cardiovascular diseases, or cerebrovascular diseases; and (4) a MoCA test [23, 24] score < 15.50, in order to exclude participants with dementia.

The study conforms to the ethical principles of the Helsinki Declaration (1975, revised in 2008), with the approval from the local ethics committee (Don Carlo Gnocchi Foundation, Milan). Informed written consent was obtained from all participants before the study began.

### 2.2. Procedure

After being consecutively recruited, all aMCI subjects underwent three steps of evaluation, six months apart (T0; T1; T2), complying with the timing of the real clinical-rehabilitative setting. In the first step of evaluation (*first step of evaluation*, T0), they were subjected to a conventional neuropsychological and ToM assessment to obtain their global cognitive level and to evaluate their affective and cognitive ToM profile at the baseline. Subsequently, they were tested 6 months (*second step of evaluation*, T1) and 12 months (*third step of evaluation*, T2) after the baseline. To avoid learning effects, ToM tests were not administered at 6 months. Between the *first* and the *second* steps of evaluation, the aMCI participants underwent a 6-week MST@H following the “Multidimensional Stimulation Therapy” (MST) model proposed by Baglio et al. [26] and adapted for MCI [28, 29].

### 2.3. First Step of Evaluation: Neuropsychological and ToM Measures

All aMCI and HC subjects were evaluated and compared at the baseline with a conventional neuropsychological and ToM battery administered by a trained neuropsychologist blinded towards the multidimensional intervention.

Among the neuropsychological measures, the Montreal Cognitive Assessment (MoCA) [23] was administered to assess the global cognitive level through several subtasks: memory (M), visuospatial abilities (VSP), executive functions (EF), attention (ATT), language (L), and temporal/spatial orientation (OR). Adjusted and equivalent scores for the total MoCA score and for each cognitive domain subscores were provided according to the normative data in the Italian population sample [24]. One of the three available parallel forms of this test [30] was randomly administered in order to avoid learning effects in the following steps of evaluation.

ToM ability was investigated at different levels of complexity and from both cognitive and affective point of view with a selection of tasks traditionally applied in research on adult and elderly subjects. The battery includes:

- the *Deceptive Box task* (DB) [31–33], which was administered as a baseline measure of ToM competences. In this first-order false belief task, participants are shown a closed box of sweets which actually contains staples rather than candies. Subjects are asked what the closed box contains before showing the real content. Then, the box is closed again, and the participants are asked what another person, who has not seen inside the closed box, will think is inside (first-order false belief question). Participants are also asked to say what they have thought about the content before opening the box (first-order own false belief question). Two control questions are also provided. Each question is scored 1 if the answer is correct and 0 if the answer is wrong (range 0-5);
- the *Reading the Mind in the Eyes test* (RME test) [34] conceived by Baron-Cohen et al. [35] to assess the attribution of affective mental states. It consists of 36 black-and-white photographs showing the eye region of different human faces, either male or female. Participants have to choose which word best describes what the person is thinking or feeling from four mental state terms written under each picture. A glossary for each term was available to participants in order to minimize comprehension difficulties, and an example was provided at the beginning of the task to familiarize subjects with the material. The Gender Test was used as a control condition assessing a basic visual face discrimination ability such as gender attribution. Each item is scored 1 if the answer is correct and 0 if the answer is wrong (range 0-36);
- a selection of four stories from the *Strange Stories* task (SS task) [36] to assess a more advanced level of ToM reasoning about the social world, which refers to cognitive ToM. In the Strange Stories task, four mentalistic stories were read to the participants consequently. At the end of each story, participants were asked three questions: a comprehension question, a mentalistic question, and a justification one. Four physical stories were also used as a control condition, in order to assess the understanding of physical events and check the presence of any comprehension deficit. The physical control stories had just one question. Each question received a score of 0 for wrong answers, 1 for partially correct/incomplete answers, and 2 for correct answers (range 0-2 for each question). The global scores of the four “ToM stories” and of the four physical stories ranged from 0 to 8.

Subjects with no more than one mistake to the control questions in the SS task were included in the analysis. The performance of the control tasks (the Gender Test for the RME test and the physical stories for the SS task) was also considered.

### 2.4. Second Step of Evaluation

**Figure 1:**
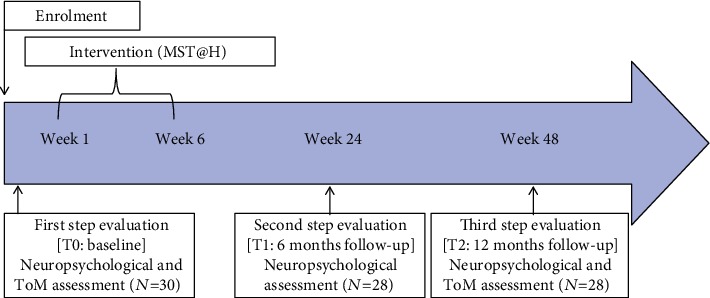
Timeline of the longitudinal study. MST@H = multistimulation treatment at home program.

Between the T0 and the T1, all aMCI participants took part in MST@H (Figure 1). Such intervention consists of 30 home-based rehabilitation sessions over 6 weeks according to the “Multidimensional Stimulation Therapy” (MST) model proposed by Baglio and colleagues [26] and adapted for people with MCI [28, 29]. The training involves daily cognitive activities (5 days a week), light aerobic physical activities (7 days a week), and occupational/recreational activities aimed at promoting the patient-caregiver interaction at home (7 days a week). The paper-pencil cognitive activities were designed to reinforce multiple cognitive domains, such as memory, attention, executive functions, language, and visuospatial abilities. The motor activity consisted of a 30-minute walk, once a day, to be carried out at any time of the day. Finally, the occupational/recreational module includes suggestions of social activities to be carried out with the caregiver during the weekend, such as watching a movie, gardening, and cooking.

Before starting the program, the researcher provided participants and their caregiver with a brief training session including instructions on the number of days per week in which to perform the cognitive and motor exercises. Phone contacts from the care manager were also planned during the six weeks of intervention to support the patient-caregiver dyad and to verify the adherence to the training program.

The aMCI group performed the second step of evaluation after 6 months from the baseline (T1) using a parallel form of the MoCA test, in order to avoid learning effects [30].

### 2.5. Third Step of Evaluation

In the third step of evaluation 12 months after the baseline (T2), the aMCI subjects underwent both the neuropsychological assessment, using a parallel form of the MoCA test [30], and the ToM evaluation, with the same battery proposed at T0. The rate of clinical conversion from MCI to AD was also collected at T2 evaluation.

### 2.6. Data Analysis

All statistical analyses were conducted using the IBM SPSS Statistics software, version 24. Descriptive statistic included frequencies for categorical variables and Means and Standard Deviation (SD) for continuous measures.

To test the level of cognitive and affective ToM competences in people with aMCI, compared at T0 with a group of HC, we used independent two-sample *t*-test (two-tailed with *p* value < 0.05 was considered statistically significant).

In order to monitor ToM abilities over time, a Repeated Measure ANOVA was performed within the aMCI group. The within-subject factors were summarized with Mean and Standard Deviation. In the pairwise comparisons, the different measurements were compared to each other and LSD *post hoc* correction for multiple comparisons was applied for *p* values. Results have been considered as statistically significant when surviving *p* corrected < 0.05 threshold.

We computed Spearman correlation analyses and linear regression analyses to explore the relationship between ToM performance and cognitive functioning over time.

A partial correlation analysis (with age, educational level, and delta MoCA scores considered as nuisance covariates) was also conducted in the aMCI group in order to test the relationship between RME changes (T2 vs. T0) and SS changes (T2 vs. T0). According to the differences between T2 and T0 (T2 vs. T0) on the MoCA test (conventional clinical measure of disease progression on cognitive domain), the aMCI group has been split into two subgroups: the MoCA+ (MoCA T2 vs.T0 ≥ 0) subjects and the MoCA- (MoCA T2 vs.T0 < 0) subjects. An independent two-sample *t*-test was computed between these two groups (MoCA+; MoCA-) on changes (T2 vs. T0) in neuropsychological tests and ToM tasks. A statistical threshold of *p* < 0.05 was considered statistically significant.

## 3. Results

### 3.1. Neuropsychological and ToM Assessment at Baseline: Comparison between MCI and HC Groups

Table 1 reports the demographic and clinical characteristics of the sample. aMCI and HC subjects showed similar demographic characteristics with no significant differences in age (*t* = 1.67, *p* > 0.05), educational level (*t* = −0.776, *p* > 0.05), and gender (*X*^2^ = .25, *p* > 0.05). The global cognitive level (MoCA score) was significantly poorer in the aMCI group compared to the HC group (*t* = −9.69, *p* < 0.001).

Table 2 reports the scores (mean ± SD) of the neurpsychological assessment (the MoCA test with its specific cognitive domain subscores) and ToM tasks (the DB task, the SS task, and the RME test) for each group. The comparison between the aMCI group and the HC group showed a significant difference in the total score of the MoCA test (*t* = −9.69, *p* < 0.001) and in visuospatial abilities (*t* = −4.94, *p* < 0.001), executive functions (*t* = −5.83, *p* < 0.001), memory (*t* = −6.11, *p* < 0.001), language (*t* = −4.26, *p* < 0.001), and temporal/spatial orientation (*t* = −2.81, *p* < 0.05) subscores, but not in attention subscore (*t* = −1.65, *p* > 0.05).

As regards the ToM tasks, no differences emerged for the first-order ToM task (the DB task), which showed a ceiling effect. Regarding the advanced ToM tasks, all subjects exhibited good performance on the control tasks (the Gender Test and the physical stories), while significant between-group differences emerged on the RME test (*t* = −3.42, *p* < 0.005) and the SS task (*t* = −4.25, *p* < 0.001), with lower scores of the aMCI group compared to the HC group.

### 3.2. Longitudinal Neuropsychological and ToM Evaluation

Table 3 illustrates the descriptive data and the comparisons between each step of evaluation (T0; T1; T2) of the longitudinal analysis within the aMCI group (Repeated Measure ANOVA). Three participants out of 30 were excluded from the analyses: two subjects dropped out from the study and one subject converted into frank dementia.

The Repeated Measure ANOVA showed that some within-subject factors change over time. Specifically, significant differences emerged in the total score of the MoCA test (*F*_(2, 52)_ = 4.776, *p* < 0.05, *ηp*^2^ = 0.155, observed power = 0.771), with a better performance in T1 and in T2 compared to T0 (T0 < T1, *p* < 0.05; T0 < T2, *p* < 0.05), and in some cognitive subdomains: executive functions (*F*_(2, 52)_ = 9.782, *p* < 0.001, *ηp*^2^ = 0.273, observed power = 0.977), memory (*F*_(2, 52)_ = 3.207, *p* < 0.05, *ηp*^2^ = 0.110, observed power = 0.588), and temporal/spatial orientation (*F*_(1.63,42.3)_ = 3.927, *p* < 0.05, *ηp*^2^ = 0.131, observed power = 0.616). In particular, pairwise comparisons showed an improvement of the executive function (T1 < T2, *p* < 0.05; T0 < T2, *p* < 0.001) and memory (T0 < T1, *p* < 0.05), while orientation gets slightly worse (T1 > T2, *p* < 0.05; T0 > T2, *p* < 0.05) (Table 3).

As concerns the first level of ToM reasoning, our results showed that the first-order ToM task (the DB task) remains stable over time. Regarding the advanced ToM tasks, our results showed a significant improvement in the SS task (T0 < T2, *F*_(1, 26)_ = 7.947, *p* < 0.05, *ηp*^2^ = 0.234, observed power = 0.774), while the RME test remains stable over time (T0 = T2, *p* > 0.05) (Table 3).

### 3.3. Relationship between Longitudinal Changes in ToM Performance and Cognitive Status

We explored possible correlations between the affective/cognitive ToM performance and the cognitive performance at T0 and T2. At T0, the MoCA test was not correlated neither with the SS task (*p* = 0.342) nor with the RME test (*p* = 0.536); at T2, the MoCA test was positively correlated with the SS performance (*r* = 0.474, *p* = 0.013). The linear regression analysis also showed that the MoCA test at T2 significantly predicted the SS performance at T2 (*β* = 0.582, *t* = 3.578, *R*^2^ = 0.312, *F*_(1, 25)_ = 12.805, *p* = 0.001).

In order to better understand the longitudinal pattern of changes of ToM performance, we also correlated ToM performance across T0 and T2. Our results showed no correlations between the SS performances (*p* = 0.268), while there was a positive, significant correlation between the RME performances (*p* < 0.001). Moreover, the linear regression analysis showed that the MoCA test at T0 significantly predicted the SS performance at T2 (*β* = 0.501, *t* = 2.894, *R*^2^ = 0.221, *F*_(1, 25)_ = 8.376, *p* = 0.008).

**Figure 2:**
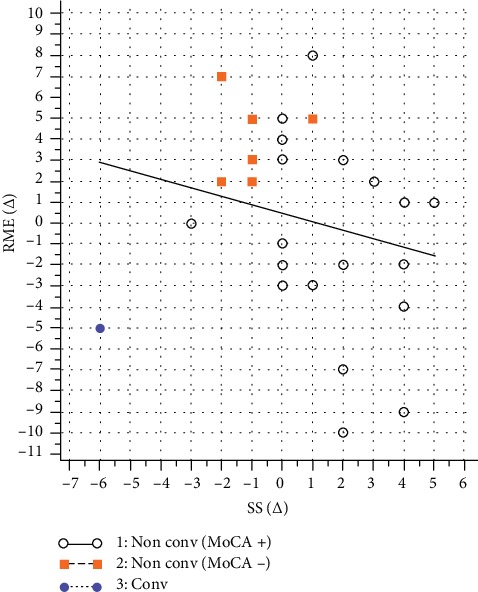
Partial correlation analysis between changes (delta score T2 vs. T0) of RME test and SS task in aMCI sample. Non conv = non converter; Conv = converter; MoCA+ = MoCA delta score T2 vs. T0 ≥ 0; MoCA- = MoCA delta score T2 vs. T0 < 0.

Finally, the result of the partial correlation analysis between changes (T2 versus T0) of affective (RME test) and of cognitive (SS task) measures of ToM showed no significant correlations (*p* = 0.065) (Figure 2). According to conventional primary cognitive outcome measure of treatment efficacy, the aMCI sample has been divided into the MoCA+ group (MoCA delta score T2 versus T0 ≥ 0) and the MoCA- group (MoCA delta score T2 versus T0 < 0). The resulted two groups were comparable for age (*t* = 0.915, *p* = 0.368), gender (*X*^2^ = 0.039, *p* = 0.843), and global cognitive level (MoCA score: *t* = −1.06, *p* = 0.301) at baseline evaluation.  Table 4 highlights a significant difference between the changes in the SS task (MoCA+ > MoCA-; *t* = 3.25, *p* = 0.003) and in the RME test (MoCA+ < MoCA-; *t* = −2.50, *p* = 0.019) between these two groups. The opposite behavior observed in the MoCA- group was pointed out also in Figure 2 (red squares).

## 4. Discussion

In this pilot study, we investigated ToM competences in AD *continuum* in a longitudinal perspective. Given the complex, multidimensional nature of ToM and the age-related difficulties on ToM abilities previously detected in this population [12, 13, 33, 37–39], we focused the attention on different levels of mentalizing reasoning (the high-level ToM competences vs. the more basic level ToM abilities) and on both cognitive and affective dimensions of ToM. Taken together, our findings highlighted the importance of the clinical assessment of ToM competences in MCI condition with different perspectives.

First of all, our preliminary results confirm the emerging evidence which consider social cognitive performance as a useful screening tool among people with neurodegenerative conditions [5]. In particular, as expected, we found that only the advanced ToM tasks are able to discriminate between aMCI and HC subjects [12, 37, 40] and that such difference concerns both cognitive and affective dimensions of ToM.

In this study, it should be noted that the ability to infer the mental states from the eye gaze was still quite preserved in our aMCI sample. In fact, despite an initial difficulty revealed from the comparison with the HC subjects in this ability, the RME scores in people with aMCI were still above the cut-off of 13. On the other hand, aMCI subjects showed an impairment in the SS performance. This result could be interpreted in the light of the cognitive load implicated in this complex ToM task, which requires relatively undamaged cognitive functions [41].

Our second main result corroborated this assumption since we found that the cognitive ToM changes (*Δ* score) were in a relationship with the longitudinal cognitive modifications in aMCI subjects. Consistent with studies in rehabilitation contexts which demonstrated the efficacy of multicomponent interventions for dementia patients on a range of outcomes, including cognition [42–44], in the present study, the MST@H seems to have a short-term impact on memory, which improved at the first step of evaluation and then tended to decrease over time, and a long-term effect on executive functions, since the observed improvement was preserved at the T2 follow-up.

Interestingly, we found that the SS performance increased over time in our aMCI sample, in relation to the enhancing of the remaining resources of aMCI individuals, such as executive functions. The relationship between cognitive ToM and executive functioning has been previously described [19, 38], but no studies investigated such relationship in a longitudinal perspective. Future studies should further deepen this issue focusing on the real-life executive functioning, for whose evaluation the only subtest of MoCA is not enough.

Several hypotheses can be advanced if we consider the results obtained by analyzing the two subgroups, MoCA+ and MoCA-, although preliminary and based on a limited number of persons per group.

The significant difference that emerged between the MoCA+ and MoCA- subjects in the SS scores seems to further support this finding, since the individuals who improve their cognitive *status* (the MoCA+ subjects) showed a related increase in their cognitive ToM performance.

Noteworthily, our third result highlighted a significant difference between the MoCA+ and MoCA- groups on the RME changes (T0 vs. T2). Interestingly, the individuals whose general cognitive functioning got worse over time (the MoCA- subjects) but who did not convert into frank dementia were the same ones who improved in the RME test. This result, although preliminary, could be explained as an unexpected long-term effect of the MST@H, which enhanced affective ToM acting on the remaining resources, such as language skills to preserve social functioning in daily life. It has been demonstrated that the RME performance is partially influenced by language skills, probably as a consequence of the verbal components of this test, but not by executive functions [45]. In our study, the language skills remain stable in people with aMCI, maybe contributing to keeping the RME performance unchanged over time. This hypothesis is corroborated by our previous work on mind-reading abilities (evaluated with the RME test) and structural connectivity changes in healthy aging [46], which demonstrated that the volume reduction at the level of premotor cortex, inferior frontal gyrus, insula and superior temporal gyrus, associated with a decrease of frontal and temporal connections, might result in difficulties to infer other's mental states through the eye gaze. However, the recruitment of additional neural circuits, such as bilateral language areas, might help to preserve the RME performance [33, 46] in elderly subjects.

To further support the role of affective ToM as a possible compensatory pathway to counteract the decline in frank dementia, future studies should consider the behavior of the MCI-converter subject, as the present study seems to suggest. Upcoming studies should investigate if changes in cognitive and/or affective dimensions of ToM in relation to a multidimensional intervention such as MST@H may be associated with changes in the neural network involved in social cognitive processes. It should be interesting to examine if interventions specifically designed to enhance affective ToM can help in maintaining the autonomy in daily life, also acting on the remaining resources of aMCI individuals. In fact, there is preliminary evidence that sociocognitive skills are sensitive to interventions specifically developed to promote ToM performance in healthy older adults [47, 48]. Further studies in rehabilitation contexts involving wider samples and comparing different types of interventions will be useful to better characterize the evolution of cognitive and affective ToM over time. In this observational study, longitudinal data have been obtained only for aMCI subjects. The next studies should also include aMCI individuals and control subjects not treated but followed longitudinally, in order to verify the trend over time of cognitive and affective ToM independently of the multidimensional intervention, and nonamnestic MCI subjects, in which the relationship between ToM skills and executive functioning can be explored in more detail. Prospective studies should also include a more extensive neuropsychological assessment for a better characterization of the cognitive profile.

Finally, a possible limitation lies in the ToM tasks selected for the evaluation of cognitive and affective mentalizing abilities. For instance, the RME test is one of most popular tests of affective ToM in the lifespan, but it is also one of the most contested tasks. In particular, it has been hypostasized that the RME test indexes emotion recognition rather than ToM ability [49]. Thus, different and more ecological ToM tasks should be used in the future to better characterize ToM performance.

## 5. Conclusion

In conclusion, our longitudinal results suggested that both cognitive and affective dimensions of ToM can be considered a useful outcome measure of a multidimensional intervention. This illustrates the crucial importance of the clinical assessment of social cognitive function in mental disorders, as strongly recommended by the American Psychiatric Association in the latest edition of the DSM-5 and highlighted also in other studies [2, 3, 5]. Furthermore, our findings demonstrated that ToM performance may constitute a valuable outcome measure of a multidimensional intervention such as MST@H, in addition to the conventional cognitive measures, which are still overly considered at the expense of interpersonal aspects, in which ToM abilities play a crucial role.

## Figures and Tables

**Table 1:** Demographic and clinical characteristics of sample at baseline evaluation (T0).

	aMCI	HC	Group comparison *p* value (^∗^)
*N* (subjects)	30	21	
Age (years; mean ± SD)	77.00 ± 4.60	74.95 ± 3.88	0.102
Education (years; mean ± SD)	10.30 ± 3.53	11.10 ± 3.70	0.442
Sex (M : F)	15 : 15	9 : 12	0.615
MoCA (total score; mean ± SD)	21.31 ± 2.36	27.93 ± 2.46	**<0.001**

^∗^Independent two-sample *t-*test (in bold the statistical significant values, *p* < 0.05). aMCI = amnestic Mild Cognitive Impairment; HC = healthy controls; *N* = number; SD = Standard Deviation; M = males; F = females.

**Table 2:** Neuropsychological and ToM results at baseline evaluation (T0).

	aMCI (*N* = 30) mean ± SD	HC (*N* = 21) mean ± SD	Group comparison *p* value (^∗^)
MoCA test			
TOT	21.31 ± 2.36	27.93 ± 2.46	**<0.001**
VSP	2.93 ± 1.02	4.10 ± 0.44	**<0.001**
EF	2.33 ± 1.17	3.95 ± 0.60	**<0.001**
M	0.53 ± 1.20	2.90 ± 1.58	**<0.001**
ATT	5.69 ± 0.83	6.07 ± 0.74	0.105
L	4.80 ± 1.01	5.83 ± 0.53	**<0.001**
OR	5.50 ± 0.83	6.02 ± 0.21	**0.002**

ToM tasks			
DB task	5.00 ± 0.00	5.00 ± 0.00	1.000
SS task	4.17 ± 1.90	6.24 ± 1.41	**<0.001**
RME test	18.27 ± 6.17	23.81 ± 4.94	**0.001**

^∗^Independent two-sample *t-*test (in bold the statistical significant values, *p* < 0.05). aMCI = amnestic Mild Cognitive Impairment; HC = healthy controls; *N* = number; SD = Standard Deviation; MoCA = Montreal Cognitive Assessment test; TOT = MoCA total score; VSP = visuospatial abilities; EF = executive functions; M = memory; ATT = attention; L = language; OR = orientation; ToM = Theory of Mind; DB = Deceptive Box task; SS = Strange Stories task; RME = Reading the Mind in the Eyes test.

**Table 3:** Neuropsychological and ToM results within the aMCI group at each step of evaluation (T0: baseline; T1: 6-month evaluation; T2: 12-month follow-up).

	Evaluation (time interval)				
	T0 mean ± SD	T1 mean ± SD	T2 mean ± SD	Time comparison *p* value (^∗^)	Pairwise comparisons *p* value (^#^)
aMCI NC (*N* = 27)					
MoCA test					
TOT	21.61 ± 2.11	22.86 ± 3.12	22.97 ± 3.50	**0.012**	**T**0 < **T**1; T1 = T2; **T**0 < **T**2
VSP	2.98 ± 1.04	3.26 ± 1.05	3.30 ± 1.18	0.318	T0 = T1 = T2
EF	2.41 ± 1.16	2.82 ± 1.03	3.27 ± 0.89	**<0.001**	**T**0 < **T**1; **T**1 < **T**2; **T**0 < **T**2
M	0.57 ± 1.23	1.21 ± 1.55	1.04 ± 1.51	**0.049**	**T**0 < **T**1; T1 = T2; T0 = T2
ATT	5.67 ± 0.84	5.61 ± 0.86	5.77 ± 0.93	0.608	T0 = T1 = T2
L	4.85 ± 1.03	4.74 ± 1.19	4.84 ± 0.93	0.876	T0 = T1 = T2
OR	5.64 ± 0.65	5.57 ± 0.88	5.21 ± 1.35	**0.035**	T0 = T1; **T**1 > **T**2; **T**0 > **T**2

ToM tasks					
SS task	4.11 ± 1.93	—	5.33 ± 1.80	**0.009**	**T**0 < **T**2
RME test	18.29 ± 6.39	—	18.63 ± 5.29	0.735	T0 = T2

aMCI C (*N* = 1)					
MoCA test					
TOT	18.53	11.53	7.53		
ToM tasks					
SS task	6	—	0		
RME test	18	—	13		

^∗^Repeated Measure ANOVA (in bold the statistical significant values, *p* < 0.05); ^#^pairwise comparisons with LSD *post hoc* correction (in bold the statistical significant values, *p* corrected < 0.05); aMCI = amnestic Mild Cognitive Impairment; *N* = number of subjects included in the analyses; SD = Standard Deviation; MoCA = Montreal Cognitive Assessment test; TOT = MoCA total score; VSP = visuospatial abilities; EF = executive functions; M = memory; ATT = attention; L = language; OR = orientation; ToM = Theory of Mind; DB = Deceptive Box task; SS = Strange Stories task; RME = Reading the Mind in the Eyes test.

**Table 4:** Results of group comparison on longitudinal changes at 12 months (T2 evaluation versus baseline evaluation (T0)) on cognitive and ToM measure.

	Group		Group comparison *p* value (^∗^)
	MoCA+ (*N* = 21) mean ± SD	MoCA- (*N* = 6) mean ± SD	
MoCA (delta score)			
TOT	2.33 ± 1.83	−2.50 ± 1.64	**<0.001**

ToM tasks (delta score)			
SS task	1.81 ± 2.02	−1.00 ± 1.10	**0.003**
RME test	−0.76 ± 4.48	4.00 ± 2.00	**0.019**

^∗^Independent two-sample *t-*test (in bold the statistical significant values, *p* < 0.05). MoCA+ = MoCA T2 vs. T0 ≥ 0; MoCA- = MoCA T2 vs. T0 < 0; *N* = number; SD = Standard Deviation; MoCA = Montreal Cognitive Assessment test; TOT = MoCA total score; ToM = Theory of Mind; DB = Deceptive Box task; SS = Strange Stories task; RME = Reading the Mind in the Eyes test.

## Data Availability

The data used to support the findings of this study are available from the corresponding author upon request.
